# Ultrasound measurement of optic nerve diameter and optic nerve sheath diameter in healthy Chinese adults

**DOI:** 10.1186/s12883-015-0361-x

**Published:** 2015-07-07

**Authors:** Han Chen, Gui-Sheng Ding, Yan-Chun Zhao, Rong-Guo Yu, Jian-Xin Zhou

**Affiliations:** Department of Critical Care Medicine, Beijing Tiantan Hospital, Capital Medical University, No 6, TiantanXili, Dongcheng District, Beijing, 100050 China; Surgical Intensive Care Unit, Fujian Provincial Clinical College, Fujian Medical University, No. 134, Dongjie Street, Gulou District, Fuzhou, Fujian 350001 China; Department of Ultrasonography, Fujian Provincial Clinical College, Fujian Medical University, No. 134, Dongjie Street, Gulou District, 350001 Fuzhou, Fujian China

**Keywords:** Optic nerve diameter, Optic nerve sheath diameter, Ultrasonography, Volunteer

## Abstract

**Background:**

Measurement of optic nerve sheath diameter (ONSD) is a fast and non-invasive method in detecting elevated intracranial pressure. However, the reported normal range of ONSD was inconsistent. The objective of the study was to determine the normal range of ONSD in healthy Chinese adults.

**Methods:**

Eyeball transverse diameter (ETD), optic nerve diameter (OND), and ONSD were measured by ultrasound examination in healthy adult volunteers. The OND and ONSD were assessed 3 mm behind the globe. The section showing maximal transverse diameter of the eyeball was frozen and the diameter was measured. Each ETD, OND and ONSD was examined twice and the mean value was calculated.

**Results:**

A total of 519 healthy volunteers were included in the study. The median (interquartile range) of ETD, OND and ONSD were 22.3 (21.6 to 23.1) mm, 3.2 (2.9 to 3.4) mm, and 5.1 (4.7 to 5.4) mm, respectively. The 95 % percentile of ONSD was 5.9 mm. There was no significant difference in ETD, OND or ONSD between male and female, or between left and right eye. ONSD was significantly correlated with OND (r = 0.62, *P* < 0.001), and the median OND/ONSD ratio (interquartile range) was 0.63 (0.59 to 0.67).

**Conclusions:**

The median and the 95 % percentile of sonographic measurement of ONSD are 5.1 mm and 5.9 mm in healthy Chinese adults. The ONSD is correlated with OND, while independent of gender, age, height, weight and ETD. The median OND/ONSD ratio is 0.63 and this parameter warrants further investigation in patients with brain injury.

## Background

Intracranial hypertension is a life-threatening syndrome, which is caused by a variety of neurological and non-neurological diseases. Catastrophic deterioration of brain function and death may occur if intracranial hypertension is left undetected and untreated [1–3]. Monitoring of intracranial pressure (ICP) is recommended in the management of intracranial hypertension [4].

Invasive intracranial devices including intra-ventricular catheters and intra-parenchymal probes are considered the gold standard for ICP measurement [5, 6]. However, invasive ICP monitoring may lead to complications including hemorrhage, infection and catheter blockage or dislodgement. Moreover, it is contraindicated in the situation of coagulopathy or thrombocytopenia [7–9]. Therefore, non-invasive ICP monitoring has been of a major interest in clinical practice [4].

Although elevated ICP can be detected by computed tomography or magnetic resonance imaging, these techniques are expensive, time-consuming and requiring patient’s transportation. In recent years, measurement of optic nerve sheath diameter (ONSD) by ultrasonography has been developed and suggested as a possible indicator of intracranial hypertension [10]. The optic nerve sheath is a membrane covering the optic nerve behind the eye, and is continuous with the dura mater over the brain. Elevation in ICP can be transmitted through the subarachnoid space, causing extension of the space. The rapid and non-invasive nature of ultrasonography results in its increasingly use for detecting elevated ICP at bedside in the emergency and critical care settings.

However, there has been no consensus on the optimal cut-off value of abnormal ONSD to indicate elevated ICP; and the existing thresholds are only from patients with brain injury. The normal range of ONSD in healthy population is indispensable information to interpret the measurement of ONSD as a marker of intracranial hypertension. Moreover, it is still unknown whether there is a difference of ONSD values between Chinese and other races, due to the lack of related studies. We carried out this prospective observational study to determine the normal range of ONSD in healthy Chinese adults.

## Method

Healthy volunteers were prospectively included in the study from October 2014 to January 2015. All subjects were aged 18 years or older. Subjects were excluded if they had a history of neurological disorders, hyperthyroidism or diseases of the optic nerve. Written informed consent was obtained from all subjects before enrollment. The study was approved by the local ethics committee of Fujian Provincial Hospital and performed according to the ethical standards of the latest Declaration of Helsinki.

**Fig. 1:**
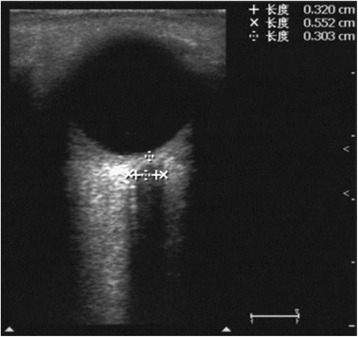
Transbulbar sonography of the optic nerve and the optic nerve sheath. OND and ONSD were measured 3 mm behind the globe using an electronic caliper along the axis. The interval between “+” marks was OND, and the interval between “x” marks was ONSD

At study entry, the gender, age, height and weight of the subject were collected. The ultrasound examination was conducted in B-mode by using a Philips Envisor C (M2540A) ultrasound system and a 12–3 MHz linear array probe (Philips Medical Systems, Bothell WA, USA), by one of the two sonographers (GS.D. and YC.Z.). Volunteers were examined in supine position with their head elevated to the angle of 20-30°. A thick layer of gel was applied over the closed upper eyelid. The probe was placed on the gel in the temporal area of the eyelid to prevent pressing the eye. The ETD, OND and ONSD were measured. OND and ONSD were assessed 3 mm behind the globe using an electronic caliper along the axis perpendicularly the retina (Fig. 1). In order to gauge the ETD, horizontal sections of eyeball were obtained by scanning from the superior to the inferior side. The section showing the maximal ETD was frozen and the diameter was measured using the electronic caliper. Each ETD, OND and ONSD was examined twice and the mean value was calculated. We also calculated the OND/ONSD ratio.

Statistical analysis was performed by using SPSS 19.0 (IBM Corporation, New York, USA). Basic descriptive statistics were calculated (including median and quartiles, mean ± SD, max, min, and 95 % percentile). Comparison of continuous variables was performed by using Mann–Whitney *U*-test. Kruskal-Wallis test was used to compare the difference of ONSD between subjects with different heights. Spearman rank correlation test was used to determine the correlation between the ONSD and other parameters including age, gender, height, body mass, OND and the ETD. Two-tailed *P* values of < 0.05 were considered to be statistically significant.

## Results

### Baseline characteristics

A total of 519 healthy volunteers were enrolled during the study period, consisting of 207 (39.9 %) males and 312 females (60.1 %). Mean (± SD) age, height and weight of the subjects were 46.1 ± 14.2 years, 163.2 ± 7.7 cm and 60.9 ± 11 kg, respectively.

### Measurements of ETD, OND and ONSD

**Fig. 2:**
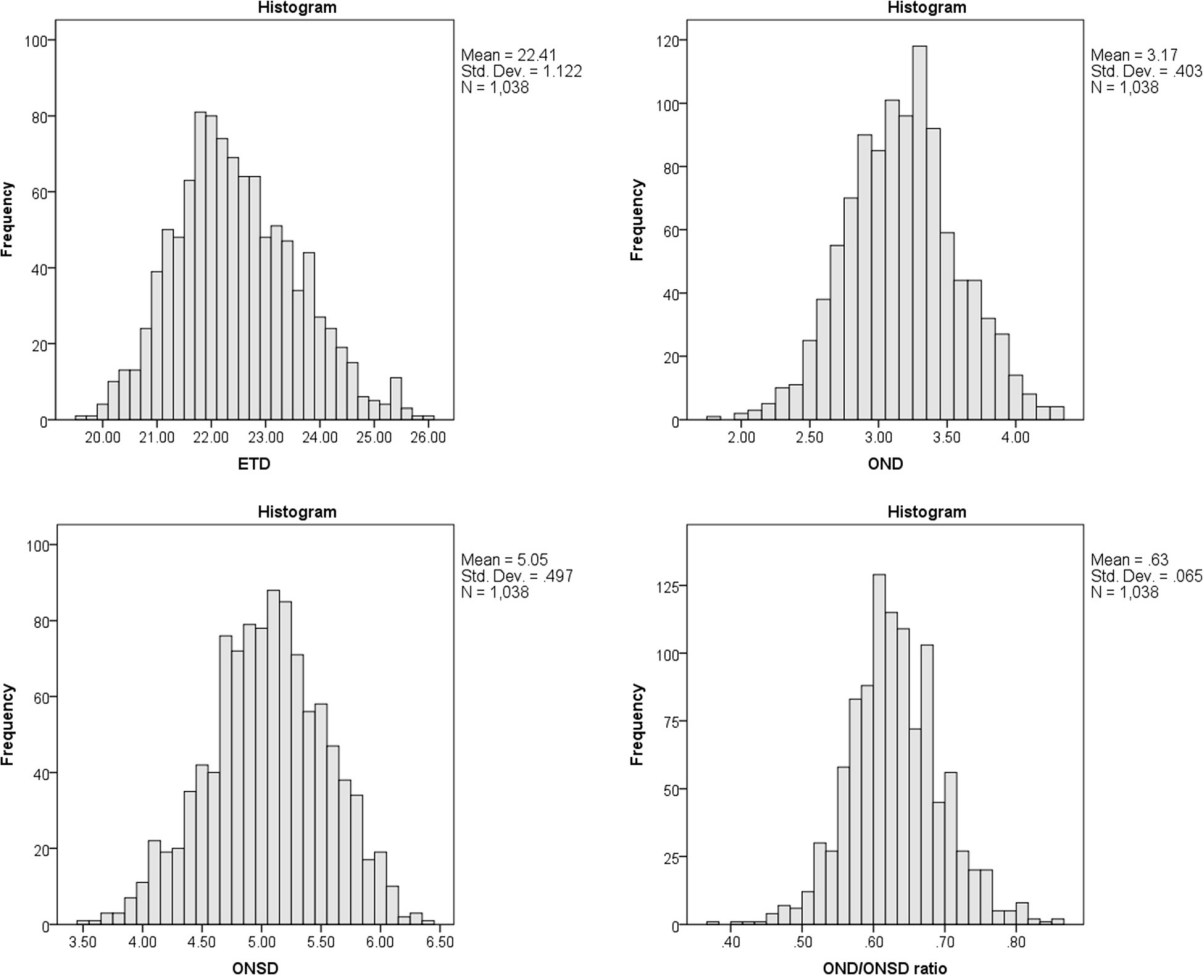
Histograms of ETD, OND, ONSD and OND/ONSD ratio. All the measurements were non-normal distributed. The ETD and OND/ONSD ratio were positive-skewed distribution while the OND and the ONSD were negative-skewed distribution

All parameters of eye measurement were non-normal distribution (Fig. 2). The median (IQR) of ETD, OND and ONSD were 22.3 (21.6 to 23.1) mm, 3.2 (2.9 to 3.4) mm, and 5.1 (4.7 to 5.4) mm, respectively (Table 1). In addition, the 95 % percentile of ONSD was 5.9 mm.

**Table 1:** Basic descriptive statistics of ETD, OND, ONSD and OND/ONSD ratio

	Mean ± SD	Median (quatile range)	Minimum	Maximum
ETD (mm)	22.4 ± 1.1	22.3 (21.6 - 23.1)	19.6	25.9
OND (mm)	3.2 ± 0.4	3.2 (2.9 - 3.4)	1.8	4.3
ONSD (mm)	5.1 ± 0.5	5.1 (4.7 - 5.4)	3.5	6.4
OND/ONSD ratio	0.63 ± 0.07	0.63 (0.59 - 0.67)	0.37	0.85

This table shows the combined results of both eyes (*n* = 1038). All the measurements were non-normal distributed

*ETD* eyeball transverse diameter, *OND* optic nerve diameter, *ONSD* optic nerve sheath diameter, *SD* standard deviation

**Fig. 3:**
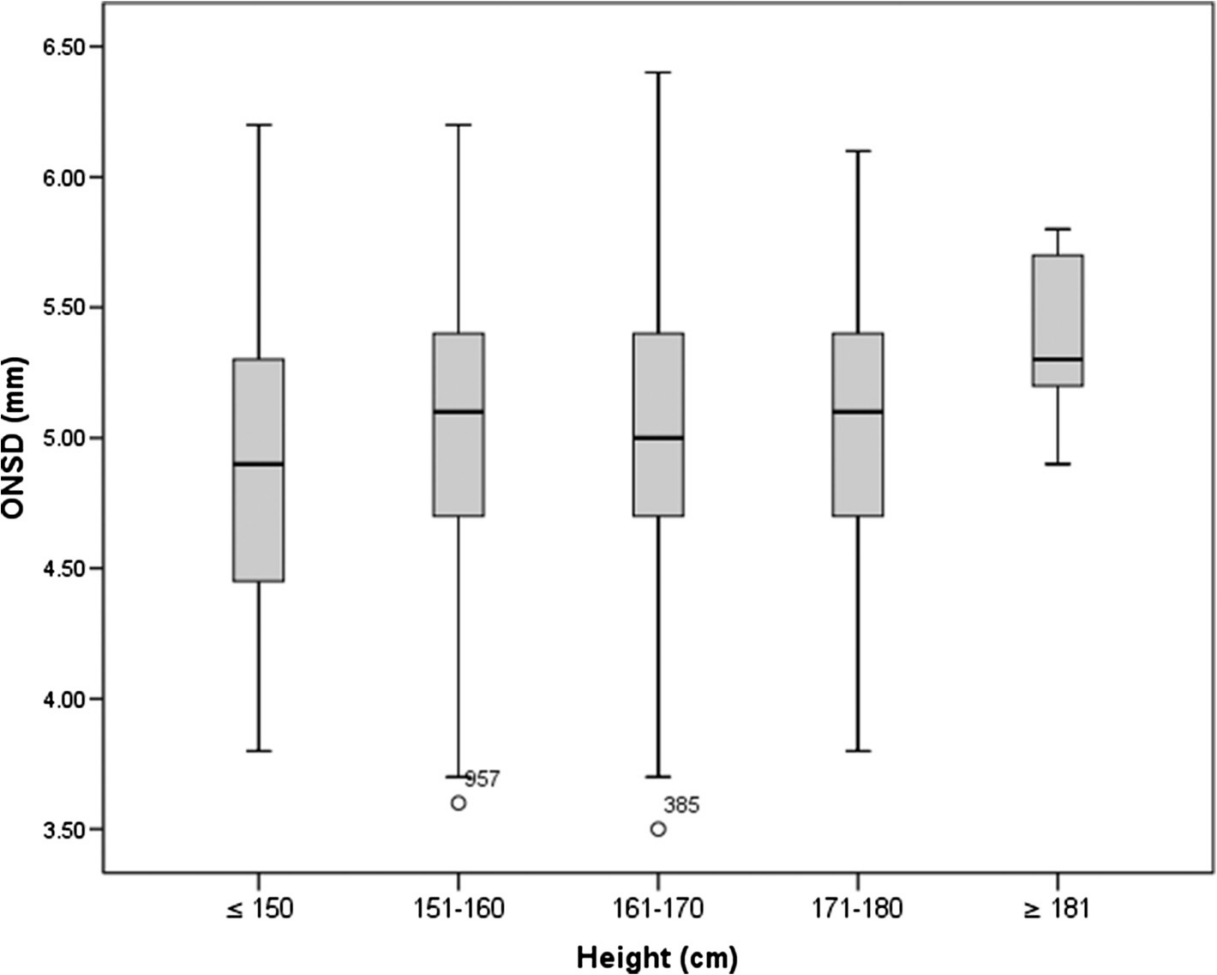
Box plot of the ONSD of different heights. Compared by Kruskal-Wallis test, *P* = 0.109

There was no significant difference in ETD, OND and ONSD between the male and female, or between the left and right eye (Table 2). ONSD was significantly correlated with OND (r = 0.62, *P* < 0.001). The median OND/ONSD ratio (IQR) was 0.63 (0.59 to 0.67). Although a statistically significant correlation was found between ONSD and height, the correlation coefficient was sub-optimal (r = 0.063, *P* = 0.041). Therefore we compared the ONSD between different height and no difference was observed (*P* = 0.109, Fig. 3). There was no linear relationship of ONSD with ETD (r = 0.044, *P* = 0.155), with age (r = 0.023, *P* = 0.467), or with weight (r = −0.005, *P* = 0.866).

**Table 2:** Comparisons of measurements between genders and between left/right eyes

	Between male and female			Between left and right eyes		
	Male	Female	*P* value	Right	Left	*P* value
	(*n* = 414)	(*n* = 624)		(*n* = 519)	(*n* = 519)	
ETD (mm)	22.3 (21.6 - 23.4)	22.3 (21.6 - 23.1)	0.208	22.3 (21.6 - 23.1)	22.3 (21.7 - 23.1)	0.894
OND (mm)	3.2 (2.9 - 3.4)	3.1 (2.8 - 3.3)	0.895	3.2 (2.9 - 3.4)	3.2 (2.9 - 3.4)	0.284
ONSD (mm)	5.1 (4.7 - 5.4)	5.1 (4.7 - 5.4)	0.233	5.1 (4.7 - 5.4)	5.0 (4.7 - 5.3)	0.334
OND/ONSD ratio	0.62 (0.58 - 0.67)	0.63 (0.59 - 0.67)	0.079	0.63 (0.59 - 0.67)	0.63 (0.59 – 0.67)	0.429

Data were presented as median and inter-quartile range

*ETD* eyeball transverse diameter, *OND* optic nerve diameter, *ONSD* optic nerve sheath diameter

## Discussion

In this large-sample-size study we identified the normal range of ETD, OND, ONSD and OND/ONSD ratio in Chinese adult population. The main findings were: the ONSD were correlated with OND; the median (IQR) ONSD was 5.1 (4.7 to 5.4) mm and the median OND/ONSD ratio (IQR) was 0.63 (0.59 to 0.67).

The measurement of ONSD by ultrasonography is a rapid, noninvasive and repeatable technique, and the result is not affected by change in position [11]. Moreover, previous studies demonstrated a good intra- and inter-observer reliability of the measurement of ONSD by ultrasonography [12, 13]. It has been suggested as an indicator of intracranial hypertension in recent years [10, 14–19]. However, there is still no unified standard threshold of enlarged ONSD to determine intracranial hypertension, which varies from 5.0 mm to 5.9 mm [20].

In studies of healthy volunteers, the range of ONSD measured by ultrasonography also varied obviously. Ballantyne et al. reported that the mean ONSDs measured by 3 observers were 3.2, 3.4 and 3.6 mm, respectively [13]. However, after carefully inspecting the method of measurement in this study, we found that the authors actually reported the OND value, rather than the ONSD [13]. In Romagnuolo et al.’s study conducted in the USA, the mean ONSD varied from 4.4 to 4.8 mm in different positions [11]. Maude et al. reported that median ONSD was 4.41 mm with 95 % of subjects in the range of 4.25 to 4.75 mm in Bangladesh [21]. While Bauerle et al. reported that the mean ONSD was 5.4 mm with a range of 4.3-7.6 mm in German [12].

Compared to previous ultrasonographic studies, we found that our data of OND was similar to Ballantyne et al.’s study, while slightly smaller than Bauerle et al.’s study; the ONSD was greater in our study than Maude et al.’s and Romagnuolo et al.’s studies. The study with largest sample size which measured ONSD using ultrasonography was conducted by Maude et al. in Bangladesh (*n* = 136) [21]. The median (5.1 mm versus 4.41 mm) and range of ONSD (3.5 to 6.4 mm versus 4.2 to 4.8 mm) in our study was higher than Maude et al.*’s* report, which compromising 12.5 % subjects with age under 16 years old [22]. This might be the possible explanation of the discrepancy in ONSD measurements. In Romagnuolo et al.’s study, because the authors primarily intended to detect difference with positions rather than to establish the normal range of ONSD, they only enrolled 10 subjects [11]. Data obtained from a smaller sample size may lead to greater sampling error. The difference between these two studies may be due to sampling error.

There are also studies using CT or MRI scan, which can provide high spatial resolution, to determine normal range of ONSD. Vaiman et al. reported the mean distal (3 mm behind the globe) ONSD (range) was 4.94 ± 1.51 (3.5-7.5) mm in the right eye and 5.17 ± 1.34 (3.8-7.9) mm in the left eye in CT scans with healthy Israeli volunteers [22]. Geeraerts et al. reported that the ONSD was 5.08 ± 0.52 mm in T2-weighted MRI in British volunteers [23]. The results of these studies were similar to ours. Previous study had shown a good correlation and agreement between ultrasound and MRI measurement of the ONSD 3 mm behind the papilla [24]. However, it is unknown whether it is comparable between CT and ultrasound measurement of ONSD. While Bauerle et al. reported the ONSD was 5.69 ± 0.77 mm in MRI scan in German [24]. Another study conducted by same authors using ultrasonography also exhibited greater ONSD value in German [12]. Therefore we assume that the difference may be caused by difference of ethnicities.

We also compared our data to the reports of elevated ICP patients. The 95 % percentile of ONSD in our healthy volunteer study was 5.9 mm; whereas the lower bound of 95 % confidence interval of ONSD in those patients with elevated ICP (with a ICP value ≥ 20 mmHg) were ≥ 5.9 mm in most of the studies [14, 17–19, 25, 26], except Kimberly et al.’s study (approximate 5.1 mm) [16]. Our data suggested that 95 % of normal Chinese adults have an ONSD value ≤ 5.9 mm and it is reasonable to speculate that an ONSD value > 5.9 mm could be considered as abnormal.

We found that OND correlated with ONSD. It is known that the ONSD is strongly correlated with increased ICP while the OND is not [15]. Since the optic nerve is part of the central nervous system, which is surrounded by a subarachnoid space, elevation in ICP can be transmitted through the subarachnoid space and cause extension of the space (e.g. increased ONSD). The change of correlation of OND with ONSD will occur, and therefore the OND/ONSD ratio might be useful in detecting intracranial hypertension. To the best of our knowledge, there is no study report the OND/ONSD ratio. Our data indicate that OND/ONSD ratio had a relatively narrow normal range and maybe a promising surrogate of ONSD. Our research provides reference data for further investigations in patients with elevated ICP.

Our data suggested that the ONSD and the height were statistically correlated. However, a *P* value < 0.05 only indicates the rejection of the null hypothesis (rho = 0), a correlation coefficient of 0.063, although statistically significant, was too low and should be considered independent in clinical interpretation. Moreover, we stratified subjects based on different height and the subgroup analysis demonstrated no difference of ONSD between different heights.

This study had several limitations. First, a direct measure of intracranial hypertension was not included thus intracranial pressure was presumed to be normal by taking history. Second, two investigators measured the ONSDs in different patients; therefore it was not possible to determine inter-observer variability in this study. Furthermore, this study included only adult volunteers, and thereby it is unable to determine the normal range of ONSD in pediatrics.

## Conclusions

The median (IQR) sonographic measurement of ONSD is 5.1 (4.7 to 5.4) mm at 3 mm behind globe and the 95 % percentile of ONSD is 5.9 mm in healthy Chinese adults. The ONSD is correlated with OND, while independent of gender, age, height, weight and ETD. The median OND/ONSD ratio (IQR) was 0.63 (0.59 to 0.67), and this parameter warrants further investigation in population of patients with brain injury.

## Abbreviations

ONSD: Optic nerve sheath diameter
ETD: Eyeball transverse diameter
OND: Optic nerve diameter
IQR: Interquartile range
ICP: Intracranial pressure
SD: Standard deviation
